# Transscleral Delivery of Dexamethasone-Loaded Microparticles Using a Dissolving Microneedle Array

**DOI:** 10.3390/pharmaceutics15061622

**Published:** 2023-05-30

**Authors:** Rawan Fitaihi, Shorooq Abukhamees, Mine Orlu, Duncan Q. M. Craig

**Affiliations:** Research Department of Pharmaceutics, School of Pharmacy, University College London, 29-39 Brunswick Square, London WC1N 1AX, UK; rfitaihi@ksu.edu.sa (R.F.); sh.abukhamees@hu.edu.jo (S.A.); m.orlu@ucl.ac.uk (M.O.)

**Keywords:** dissolvable microneedle, ocular microneedle, dexamethasone, ocular delivery, transscleral permeation, PLGA microparticles

## Abstract

Microneedles (MNs) have attracted considerable interest as a means of ocular drug delivery, a challenging delivery route due to the limitations imposed by the various biological barriers associated with this organ. In this study, a novel ocular drug delivery system was developed by formulating a dissolvable MN array containing dexamethasone-loaded PLGA microparticles for scleral drug deposition. The microparticles serve as a drug reservoir for controlled transscleral delivery. The MNs displayed sufficient mechanical strength to penetrate the porcine sclera. Dexamethasone (Dex) scleral permeation was significantly higher than in topically instilled dosage forms. The MN system was able to distribute the drug through the ocular globe, with 19.2% of the administered Dex detected in the vitreous humour. Additionally, images of the sectioned sclera confirmed the diffusion of fluorescent-labelled microparticles within the scleral matrix. The system therefore represents a potential approach for minimally invasive Dex delivery to the posterior of the eye, which lends itself to self-administration and hence high patient convenience.

## 1. Introduction

Ocular diseases may vary from simple self-limiting conditions, such as conjunctivitis, to more complex diseases associated with irreversible visual impairment [1]. Diseases of the posterior segment, such as wet and dry age-related macular degeneration, diabetic macular oedema, cytomegalovirus, proliferative diabetic retinopathy, endophthalmitis, and uveitis are the most prevalent among such conditions [2]. In order to treat posterior segment diseases, an effective delivery strategy must be employed due to the inaccessibility of the target tissue. Topically applied medications, such as drops and ointments, suffer from extremely low bioavailability (<5%) [3], which is attributed to the anatomical and physiological mechanisms of drug elimination, including tear fluid turnover, the blinking reflex, and tear drainage. Therefore, large and frequent doses are required to achieve effective concentrations [4]. Similarly, the systemic route requires the administration of a large dose to deliver an effective concentration at the target site; hence, off-target adverse effects are a distinct possibility. Intraocular injection, on the other hand, can directly deliver a high dose at the target site. However, along with patient inconvenience, the intravitreal route has been associated with serious side effects, including infection and retinal detachment [5]. Thus, an alternative, efficient, localised, and non-invasive approach is highly desirable.

Microneedle (MN)-based systems have emerged as a minimally invasive method to enhance drug delivery. While this approach has been primarily designed for transdermal uses, new applications of microneedles have recently been developed, including the delivery and localisation of therapeutic agents within ocular tissue. These systems can be utilised to deliver a wide range of therapeutics due to their minimally invasive nature, while also penetrating the ocular barrier for site-specific drug localisation [1]. Ocular applications of MNs have been focused mainly on the use of hollow, solid, and dissolvable MN systems [6]. Dissolvable MNs represent a patient-friendly drug delivery system that may potentially be self-applied. These MNs are fabricated using dissolvable hydrophilic and biodegradable polymers. However, a relatively limited number of studies has been conducted to investigate their feasibility for ocular delivery [7,8,9,10,11,12,13,14].

Polylactic-glycolic acid (PLGA) is biocompatible, biodegradable polymer that has been commonly employed to fabricate several systems for ocular delivery purposes, either alone or in combination with other polymers, typically to produce controlled release vehicles [15,16]. PLGA offers a tuneable degradation profile by altering the lactide to glycolide ratio [16]. Several PLGA-based drug delivery systems have been prepared to target both the anterior and posterior segments of the eye, with hydrophilic and hydrophobic payloads ranging from small molecules such as sparfloxacin [17] and fluocinolone acetonide [18] to macromolecules such as bevacizumab [19], infliximab [20], and ranibizumab [21]. Several approaches have been developed to deliver drugs to the eye utilising PLGA, including using micro- and nanoparticles incorporated into topical ophthalmic preparations [17,22] or intraocular injections [23], as well as contact lenses [24], ocular films [25], implantable devices including the commercially available Ozurdex^®^ and Durysta™ [26,27]. More recently, drug-loaded PLGA has been incorporated into dissolvable MNs [14]. Moreover, PLGA is versatile enough to allow for a relatively shorter release time, as utilised in the commercially available product Surodex™, an intraocular, biodegradable implant that acts over the course of 7–10 days [28], to the longer release profile of up to 6 months of the Ozurdex^®^ ocular implant [26].

Dexamethasone (Dex) is one of the most prescribed corticosteroids for ocular delivery [29]. It is commonly used to treat ocular inflammation in the anterior and posterior segments, such as conjunctivitis, uveitis, and macular oedema [30]. The commercially available forms of Dex in ocular dosages include topical solutions, topical suspensions, ointments, intraocular injections, and ocular implants [29]. However, each of these approaches carries disadvantages in terms of bioavailability (particularly to the posterior segment of the eye) and/or patient convenience [1]. To mitigate these disadvantages, approaches such as the inclusion of spreading enzymes, such as recombinant human hyaluronidase, have been reported to improve the delivery of drugs or drug carriers to the posterior segment [31,32,33].

Nevertheless, the development of a minimally invasive system which allows significant drug delivery to the posterior segment while also allowing patient self-administration would be of great interest.

Here, we describe a system whereby Dex is loaded into PLGA microspheres which are in turn loaded into MN systems, with the aim of developing a delivery approach which would allow the slow release of Dex to the posterior of the eye while also having the potential for self-administration. We have focused on Dex in this study as it has a potent anti-inflammatory effect and only a very low concentration is required to treat the eye locally, rendering it a suitable candidate for MN drug delivery [34]; however, this approach can be considered to be a platform technology which is potentially applicable to several other therapeutic agents.

We utilise a dissolving MN system, composed of an MN array attached to a square base to create a patch with a potential for self-administration. The embedded PLGA particles can offer sustained drug delivery and hence improved bioavailability [35]. While particulate delivery to ocular tissue by topical or intraocular injection has been widely reported, low residence time and significant side effects have also been reported [17,29]. We therefore intend for the system described here to be a novel means of delivering Dex to the posterior of the eye, which combines the advantages of potential self-administration, lower delivery-related ocular damage, infrequent dosing, and favourable bioavailability.

## 2. Materials and Methods

### 2.1. Materials

PLGA Purasorb^®^ PDLG 7502 was obtained from Corbion Purac Biomaterials, Netherlands. Dichloromethane, methanol, trifluoroacetic acid, dexamethasone, polyvinyl alcohol, Tween 80, and sodium azide were purchased from Sigma-Aldrich, Saint-Quentin-Fallavier, France. Acetonitrile was purchased from Fisher Scientific, Loughborough, UK. Coumarine-6 was purchased from Acros Organic, Morris Plains, NJ, USA. Polyvinyl pyrrolidone Kollidon^®^30 and Kollidon^®^F90 grades were purchased from BASF, Heidelberg, Germany. Polyvinyl alcohol, gelatine, phosphate buffer saline tablets (pH 7.4), acetonitrile, and 10% neutral-buffered formalin were purchased from Sigma Aldrich, Burlington, MA, USA. Methylprednisolone was purchased from Cambridge Bioscience, Cambridge, UK. SlowFade™ gold antifade mountant and optimal cutting temperature compound (OCT Cellpath) were purchased from ThermoFisher, Oxford, UK.

### 2.2. Methods

#### 2.2.1. Preparation of PLGA Microparticles

PLGA microparticles were prepared using an oil-in-water (o/w) emulsion solvent evaporation method. To prepare the blank PLGA microparticles, 140 mg of PLGA was dissolved in 5 mL of dichloromethane (DCM). The organic DCM/PLGA solution was added dropwise to 15 mL of 1% PVA solution, while subjected to homogenisation at 18,000 rpm for 60 s using a high-speed homogeniser (Yellow line DI 18 basic, Ika, Staufen, Germany) to produce the PVA-stabilised o/w emulsion. The homogenisation of the dispersion was conducted on an ice bath. The emulsion was added to 250 mL of 0.1% PVA solution and stirred with a magnetic stirrer at 600 rpm for 4 h to evaporate the organic solvent at room temperature. Thereafter, the dispersion was washed by centrifugation at 9000 rpm for 15 min using a sigma centrifuge (Sigma 3–16K, Hertfordshire, UK) in three cycles. The supernatant was removed each time and replaced with deionized water to remove the excess PVA solution. The PLGA microparticle dispersion was then pre-frozen (−80 °C) for 12 h and subsequently lyophilized using (VirTis Advantage, SP, Stone Ridge, NY, USA) for 48 h with hold and ramp cycles from −40 °C to 20 °C. For the preparation of fluorescent-labelled PLGA microparticles, 0.5% *w/w* coumarin-6 was added to the organic DCM/ PLGA solution and, for Dex-loaded PLGA microparticles, 100 mg of PLGA and 40 mg of Dex were dissolved in 5 mL (9:1) DCM: methanol followed by the same steps to prepare the blank microparticles.

#### 2.2.2. Microparticle Characterisation

The mean particle size, size distribution, and polydispersity index (PDI) were measured by dynamic light scattering (Zetasizer, nano-zs, Malvern, UK). The freeze-dried PLGA particles were resuspended in deionized water and then sonicated for five minutes until a homogenous suspension was achieved. The mean particle size, size distribution, and PDI were measured for each batch of PLGA microparticles prepared. The measurements were performed in triplicates for each sample.

The magnitude of the surface charge was measured by the Zeta potential analyser (Zetasizer, nano-zs, Malvern, UK). The samples were placed in folded capillary cells with electrodes. The measurements were performed in triplicates for each sample.

The surface morphology of the PLGA particles was assessed by scanning electron microscopy (SEM). The samples were imaged by desktop SEM (Phenom Pro, St. Louis, MO, USA) at a 5 kV acceleration voltage. The images were then analysed by image processing software (ImageJ, Java 1.8.0_66, Bethesda, ML, USA). Coumarine-6 loaded particles were imaged by a fluorescent microscope with a 12 V mercury lamp (Nikon Microphot-FXA microscope, Nikon, Japan).

The XRD analysis of the free polymer, Dex, physical mixture, and Dex-loaded and blank PLGA microparticles was performed using a MiniFlex 600 benchtop X-ray diffractometer (RigaKu, Tokyo, Japan). Diffraction patterns were generated from raw data using Origin 2019 (OriginLab Corporation, Northampton, MA, USA).

To obtain an infrared spectrum of the initial material, physical mixture, and Dex-loaded and blank microparticles, 100 FT-IR spectrometers (Perkin-Elmer, Waltham, MA, USA) were used. Each sample was scanned 16 times in the range of 4000 cm^−1^ to 500 cm^−1^ at a 2 cm^−1^ resolution. A background scan was performed before each run.

The thermal analysis of the raw materials, physical mixture, and Dex-loaded and blank PLGA particles was performed using a Discovery TA Q2000 differential scanning calorimeter. Data collection was performed from 0 °C to 300 °C at a heating rate of 10 °C/min and under a nitrogen flow rate of 50 mL/min. An empty aluminium pan was used as a reference standard. The data were analysed using TRIOS data analysis software (TA instruments, New Castle, DE, USA). The equipment underwent routine calibration procedures using high purity indium.

#### 2.2.3. Dexamethasone Analytical Quantification

Chromatographic quantification was utilised to detect and analyse the concentration of Dex in the samples. High-performance liquid chromatography (HPLC) (Agilent^®^ 1200, Agilent Technologies, Santa Clara, CA, USA) equipped with a UV-visible detector, autosampler, quaternary pump, and degasser was used. A C18 HPLC reverse column with a 5 μm particle size, 15 cm length, and 4.6 mm internal diameter (ASCENTIS^®^, Supelco, Bellefonte, PA, USA) was used for the analysis. A gradient mobile phase of acetonitrile and acidified water (0.1% TFA water, pH 2.1) was used. The run started using a 30:70 acetonitrile to 0.1% TFA water ratio for 3 min (0–3 min), followed by a gradual change in the ratio to 70:30 acetonitrile:0.1% TFA water over 12 min (3–15 min). Thereafter, the ratio was returned to the initial gradient (30:70 acetonitrile:0.1% TFA water) over 3 min (15–18 min) and stabilised for 5 min (18–23 min). Methylprednisolone was used as an internal standard and a flow rate of 1 mL/min was maintained at 25 °C. The injection volume was 10 µL and the UV detection wavelength was set at 240 nm. The data acquired were analysed and processed with Openlab software (Agilent Technologies, Santa Clara, CA, USA). The ratio of the peak area of IS to Dex was linear over the range of 5–150 µg/mL (R^2^ = 0.999).

#### 2.2.4. Encapsulation Efficiency and Drug Loading

To determine the encapsulation efficiency and drug loading of the Dex-loaded microparticles, an accurate weight of Dex-loaded microparticles was completely dissolved in acetonitrile. Samples from the solution were filtered and analysed by HPLC to determine the amount of Dex in the particles.

The percentage encapsulation efficiency (*EE*%) and drug loading (*DL*%) were calculated by the following equations:

$$EE\%=\frac{Actual\;amount\;of\;Dex\;in\;the\;particles}{Theorical\;Dex\;amount}\times 100$$

$$DL\%=\frac{Actual\;amount\;of\;Dex\;in\;the\;particles}{total\;amount\;of\;the\;particles}\times 100$$

#### 2.2.5. Dexamethasone In Vitro Release Study

The in vitro release of Dex from the PLGA particles was performed using a modified release study. In a 15 mL falcon tube, 10 mg of the Dex-loaded particles were added with 10 mL of preheated (37 °C) PBS containing 0.05% Tween 80 and 0.02% sodium azide. The tubes were placed in an incubator shaker (Sciquip, Wem, UK) at 37 °C and 100 rpm. At specified time intervals, the tubes were centrifuged for 5 min at 5000 rpm; then, a 1 mL sample was withdrawn from the dissolution medium and replaced with a fresh buffer. The samples were kept at −20 °C until the end of the experiment. The amount of Dex was quantified by HPLC. The release was analysed in triplicates and the mean ± standard deviation (SD) was reported.

#### 2.2.6. Preparation of Microneedle Array

To prepare the MN array, a mould with 9 (3 × 3) conical needles, with 900 µm height, 450 µm base diameter and 1 mm tip-to-tip spacing on a 4 × 4 mm^2^ square base were 3D printed with an SLA 3D printer (Form 3, Formlab, Somerville, MA, USA). Polyvinyl pyrrolidone (PVP) Kollidon^®^F90 and polyvinyl alcohol (PVA) blends were used to prepare the array. Two ratios of PVP to PVA were tested (Table 1). The mould was filled with a specified volume of the polymeric solution, the moulds were then centrifuged in a rotor for 15 min at 9000 rpm, and were then left to dry for 24 h under ambient conditions. After completely drying, the MN array was gently separated from the mould.

For the preparation of PLGA particles incorporated in the dissolvable MN array, the microparticles were accurately weighed and mixed with 10% (*w*/*v*) PVP (Kollidon^®^ 30) solution with a magnetic stirrer for 10 min until the particles dispersed in the solution. A volume equivalent to 100 µg of particles was placed in each mould and centrifuged for 15 min at 9000 rpm, and was then left to dry for 24 h under ambient conditions. Next, the moulds were filled with PVP/PVA solution and centrifuged for 10 min, and were then left to dry for another 24 h under ambient conditions. The arrays were gently removed from the moulds. The MN arrays were stored in a light-protected airtight container until further use.

#### 2.2.7. Dissolution of the Polymeric Base

To test the dissolution of the fabricated polymeric MN arrays, solidified gelatine was used as a model for the sclera. Gelatine powder was added to deionised water to prepare a 15% (*w*/*v*) solution. The solution was gently heated to 70 °C, until it completely dissolved. The solution was poured into a petri dish and left to cool. The MN array was inserted in the solidified gelatine solution using gentle pressure for 15 s, left in the gelatine for 15 s, and was then removed by a tweezer and the dissolution was determined by calculating the percentage of height reduction in the MN array by taking images before and after insertion.

#### 2.2.8. Mechanical Testing

To assess the mechanical properties of the MN arrays, an Instron tensile tester 5567 (Instron, Norwood, MA, USA) was used. The MN array was fixed to the lower frame of the instrument by double-sided tape. A compression force was applied using a flathead stainless-steel cylindrical probe attached to the upper frame, vertical to the MN array, at a speed of 0.1 mm/s. The compression force was continuously recorded until a displacement of 500 μm was reached. Data were collected and analysed by Bluehill 2 (version 2.35) software. The force vs. displacement curve was constructed by Origin pro software (2019b, Originlab, Northampton, MA, USA) and was used to determine the force required for 500 µm of displacement. Data are reported as mean ± SD (*n* = 3).

#### 2.2.9. Preparation of Ocular Tissue

The ex vivo experiments, including the permeation, insertion, and diffusion tests, were conducted on excised ocular tissue. Fresh porcine eye globes were purchased from Fresh Tissue Supplies (Etchingham, East Sussex, UK). The eye’s surface was cleaned, and the remaining rectus muscles were carefully removed. The cornea was cut out around the limbus using surgical scissors. Then, the vitreous humour was removed, and the retinal layer was gently detached. A square sample of scleral tissue (~7 × 7 mm) was cut. All the scleral samples were cut at a 5 mm distance from the lumbus to minimize variation in sample thickness [7]. This location is where the subconjunctival injection is usually applied, and it is the visible part of the sclera where self-implantable MN arrays can be inserted. All the ocular tissue samples were used immediately or frozen in a sealed container at −20 °C for further use. At the time of use, the tissues were thawed and soaked in phosphate buffer saline (PBS) (pH 7.4) for 30 min at 37 ± 0.5 °C.

#### 2.2.10. Microneedle Scleral Insertion Test

The insertion force study was performed on isolated scleral tissue samples using an Instron tensile tester 5567 (Instron, USA). The scleral sample was hydrated before the experiment for 30 min in PBS (pH 7.4) at 37 ± 0.5 °C. The scleral samples were pat-dried with a paper towel and then affixed to the lower frame of the Instron tester using cyanoacrylate adhesive glue. The MN array was mounted on a cylindrical stainless-steel probe with double-sided adhesive tape, perpendicular to the tissue sample. The probe was set to move at a rate of 10 µm/s until a force of 4 N was reached. The compression force exerted on the ocular tissue by the MN array as a function of its compression distance (mm) was continuously recorded using Bluehill 2 (version 2.35) software. The tissue insertion force was determined from the distance vs. force curve as the average force when the curve showed a rapid increase in force as the MNs penetrated the tissue [36]. The test was performed in triplicates and the mean ± SD was reported.

#### 2.2.11. Microneedle Array Adhesion Test

To study the degree of adhesion of the blank polymeric array to scleral tissue, an Instron tensile tester was used. A ~7 × 7 mm square piece of scleral tissue was affixed to the lower stationary frame of the instrument, then the tissue was wetted with 20 μL PBS (pH 7.4). The MN array was attached to a moving stainless-steel probe with double-sided adhesive tape. Initially, a compression force of 4 N was applied to insert the MN array into the tissue and the probe was held in position for 10 s, and then a tensile force was applied till the MN array was completely separated from the scleral sample [37]. The force corresponding to the MN array’s detachment was identified as the adhesion force. The test was performed in triplicates and the mean ± SD was reported.

#### 2.2.12. Transscleral Permeation Study

To study the permeation of Dex through ocular tissue, excised porcine sclera was used in the test. The study was performed using a Franz diffusion cell (PermeGear Inc., Hellertown, PA, USA). The acceptor compartment volume was 3 mL with a 9 mm joint orifice diameter and a 0.64 cm^2^ diffusion area. The acceptor compartment was filled with 3 mL PBS (pH 7.4) and maintained at 37 ± 0.5 °C under continuous stirring by a magnetic bar. A piece of scleral tissue, large enough to cover the orifice, was fixed to the top of the donor compartment using cyanoacrylate glue with the episcleral layer side facing upwards. The donor compartment was fixed to the acceptor compartment by the clamp that is exposing the tissue to the donor compartment. The tissue was wetted with 10 µL PBS before adding the samples. Three dosage forms were tested for transscleral permeation: a 1% Dex suspension in deionised water, free Dex-loaded microparticles (MP-Dex), and an MP-Dex-incorporated MN array (MN-Dex). Single MN-Dex or an amount equivalent to ~100 µg of Dex in free MP-Dex or 1% Dex suspension was instilled in the donor compartment. During the experiment, the top of the donor compartment and sampling arm were covered with Parafilm^®^ to preserve fluid in the tissue and cell. Samples from the donor compartment were withdrawn at predetermined time intervals, which were later replaced by fresh PBS buffer maintained at 37 ± 0.5 °C. Collected samples were kept at 4 °C to be later analysed by HPLC. The cumulative percentage of Dex that permeated through the sclera was calculated. At the end of the experiment, the scleral tissue was washed three times with PBS, then the edges covering the orifice were cut. Subsequently, the tissue was homogenised with a PBS/acetonitrile mixture by a homogeniser. The homogenised mixture was centrifuged, and the supernatant was filtered and analysed with HPLC. Results were reported as the mean ± SD (*n* = 3).

#### 2.2.13. Ex Vivo Particle Transscleral Diffusion

To study PLGA microparticle release and diffusion from the needle tip into scleral tissue, fluorescent-labelled microparticles incorporated in the MN array were used. The MN array was inserted on a ~7 × 7 mm square piece of the sclera by applying gentle pressure for 30 s. The tissue–MN array composite was wetted with 50 µL PBS and kept in a closed container at 37 ± 0.5 °C for 1 h or 2 h. The sclera was then washed three times in fresh PBS to remove MN array residue. The tissue was fixed in 10% neutral-buffered formalin for 2 h, and was then immersed in the optimal cutting temperature (OCT) compound cryo-embedding matrix, which was then kept in dry ice for 15 min, allowing the OCT to solidify. The frozen tissue block was transversely sectioned or cross-sectioned by microtome-cryostat (HM525 NX Cryostat, Thermo Scientific, Swindon, UK) at −20 °C to 12 µm thick sections, and was then mounted on Superfrost^®^ plus slides (Thermo Scientific, Swindon, UK) [38]. The sections were either stained with haematoxylin and eosin (H&E) stain, according to the manufacturer’s protocol, or stained with DAPI for 5 min, and were then washed with PBS. The sections were left to dry under ambient conditions and were then mounted with SlowFade™ gold antifade mountant to be imaged by an optical microscope for H&E staining and a fluorescent microscope (EVOS, Thermofisher, Waltham, MA, USA) for fluorescent staining.

#### 2.2.14. Ex Vivo Dexamethasone Ocular Biodistribution

This study was designed to study ex vivo Dex release and distribution from the MN array within the ocular globe. A porcine eye either fresh or thawed from frozen was hydrated for 30 min in PBS (pH 7.4) at 37 ± 0.5 °C. The eyes were placed in 3D-printed cups designed to fit porcine eyes, maintaining their shape and hydration; 200 µL PBS was added to the cup for the latter purpose. A MN array containing Dex-loaded particles (MP-Dex) was inserted into the eye globe by applying force equal to 5 N using a movable stainless-steel probe attached to a mechanical tester (a texture analyser). After insertion, the cups were covered by Parafilm^®^ and kept at 37 ± 0.5 °C in a shaking incubator (50 rpm) for 4 h. Subsequently, the MN-array residual was removed, and the eye was washed three times in a fresh PBS tub. The vitreous humour (VH) was aspirated slowly from the centre of the eye by a 5 mL syringe attached to a 25 G needle at the equator opposite the MN array insertion site [39,40]. The eye was dissected around the limbus to isolate the anterior segment (AS), including the cornea, lens, and iris–ciliary body. At this point, any VH left was withdrawn by a needle and added to the collected VH sample. The region of the sclera where the MN array was inserted was cut. Finally, the rest of the eye globe, including the sclera (excluding the insertion site) and the retina, was stored separately.

To extract Dex from VH, the liquid–liquid extraction method was used: 5 mL acetonitrile with a known concentration of IS was added to the sample, the solution was vortexed for 60 s, sonicated for 5 min, and then the mixture was centrifuged for 15 min at 9000 rpm. The supernatant was withdrawn then the solution was dried under vacuum. The dried extract was resuspended in 0.5 mL acetonitrile and then filtered by a PTFE filter (0.2 µm), and analysed using HPLC. The anterior segment, the site of MN-array insertion and the rest of the eye globe were each homogenised with an acetonitrile and PBS mixture (1:4), with IS added to the mixture. The homogenised tissue mixture was sonicated for 10 min and then centrifuged for 15 min at 9000 rpm. The supernatant was withdrawn and left to dry under vacuum. The dried extract was resuspended in 0.5 mL acetonitrile and then filtered (PTFE 0.2 µm), and analysed by HPLC.

#### 2.2.15. Statistical Analysis

All the results are presented as the mean ± standard deviation (SD). Statistical analysis was performed using one-way analysis of variance (ANOVA) or Student’s *t*-test and a *p*-value < 0.05 was considered statistically significant. All statistical analyses were performed using Prism9 software (Graphpad, La Jolla, CA, USA).

## 3. Results and Discussion

### 3.1. Microparticle Characterization

**Figure 1:**
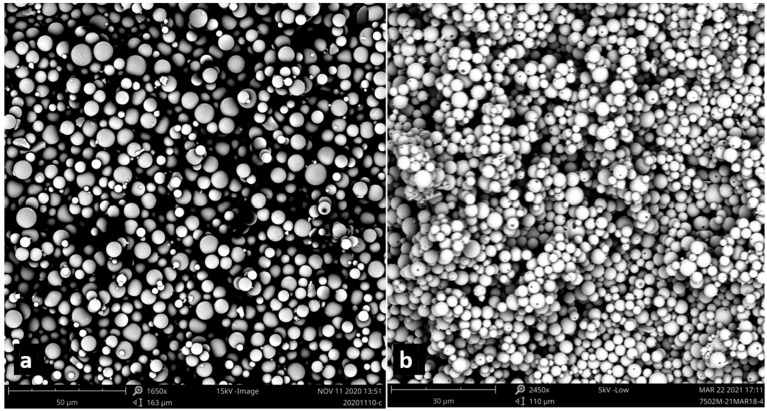
SEM images of (**a**) blank PLGA microparticles and (**b**) Dex-loaded PLGA microparticles.

Blank and Dex-loaded microparticles were prepared using an o/w solvent evaporation method. The microparticles were spherical in shape with a smooth surface, while it was noted that pinholes appeared on the surface of the Dex-loaded microparticles (Figure 1). The average size and surface charge of the microparticles are listed in Table 2. The Dex-loaded microparticles were slightly smaller than blank microparticles, possibly reflecting the higher concentration of PLGA in the organic solution, which results in a more viscous organic phase [41], although the observed differences were not statistically significant. Furthermore, the blank, fluorescently labelled, and Dex-loaded microparticles presented a negative surface charge. The surface charge was strongly influenced by the formulation composition; PVA has a negative hydroxyl group (–OH) [42], while PLGA has terminal carboxylic groups on the chains which collectively contributed to the negative surface charge values [43].

Dex is a hydrophobic drug which renders it suitable for the single emulsion solvent evaporation method [44,45,46]; however, Dex is insoluble in most of the water immiscible organic solvents. Therefore, methanol was mixed with DCM to improve Dex solubility in the organic phase [44,47]. However, the %*EE* was 49.4%, which indicates that half of the initially added Dex was lost in the fabrication process. This %*EE* is ex˃ected due to the slight solubility of Dex in water and the miscibility of the methanol with the aqueous phase, which increased the amount of Dex loss during the preparation of the emulsion. A similar %*EE* was obtained by a study that used a mixture of DCM and acetone to prepare Dex-loaded microparticles [48].

**Figure 2:**
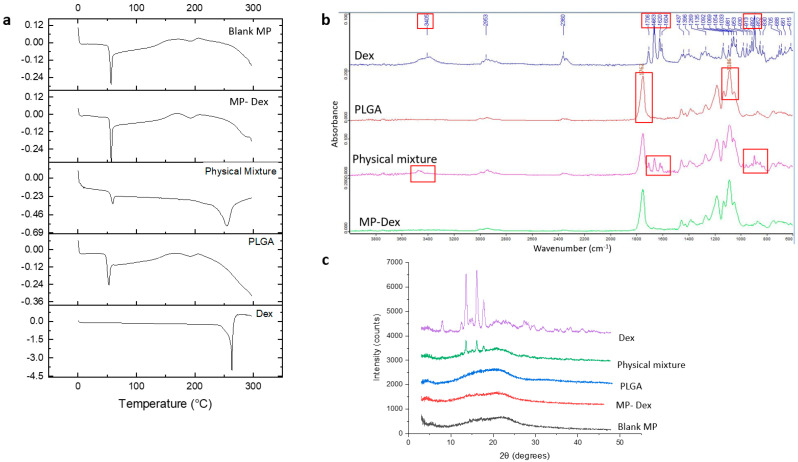
Microparticle characterization (**a**) DSC thermogram, (**b**) FTIR spectra (red boxes indicate characteristic peaks), and (**c**) X-ray diffractogram.

Dex, free PLGA polymer, physical mixtures of the drug and the polymer, and fabricated blank and Dex-loaded microparticles were assessed by DSC. The data are presented in Figure 2a. The DSC thermogram of Dex presented a characteristic sharp endothermic transition at a melting point (T_m_) of 263.5 °C, which is in accordance with the T_m_ reported in the literature [29]. PLGA powder (as received) was analysed, and a glass transition (T_g_) of approximately 53.5 °C (with endothermic relaxation) was recorded, which lies within the range of reported PLGA T_g_ values [45]. Similarly, the physical mixture thermogram exhibited two endothermic transitions at 55.5 °C and 254.5 °C, corresponding to the PLGA T_g_ and the Dex T_m_, respectively. The systems also showed a broad endotherm at circa 195 °C, which may correspond to residual solvent loss. The blank microparticles showed a thermal response that was very similar to the PLGA powder, as expected. However, the loaded microparticles showed no evidence of a melting peak for Dex; the T_g_ value was not noticeably different, although this may reflect both the low drug loading and the relative similarities of the two T_g_ values. The absence of a drug melting peak indicates that the drug may be molecularly dispersed within the microparticles.

The FTIR spectra of pure Dex, PLGA powder, the physical mixture of the drug and the polymer, and the Dex-loaded microparticles (MP-Dex) are presented in Figure 2b. From the Dex spectrum, the peak at 3405 cm^−1^ corresponds to -NH stretching vibration. Functional group peaks at 1706, 1663, 1620, and 1604 cm^−1^ are attributed to carboxyl –C=O stretching vibration linked to C3-cyclic and C20 carbonyl groups on the Dex molecule, while the C-F group axial deformation is indicated by peaks at 981 and 892 cm^−1^ [49]. The PLGA spectrum showed a distinctive peak at 1762 cm^−1^ corresponding to carboxyl –C=O stretching and a peak at 1186 for C–O stretching [50]. Dex characteristic peaks were observed in the physical mixture spectrum and not in the MP-Dex, which could be due to the low concentration of Dex in the loaded particles, and only PLGA peaks were observed where no additional peak appeared, which indicates the lack of chemical interaction between Dex and the polymer.

Diffractograms of the free Dex, PLGA polymer powder, physical mixture of Dex and PLGA, blank (blank MP) and Dex-loaded microparticles (MP-Dex) are presented in Figure 2c. The XRD diffractogram of Dex showed characteristic diffraction 2θ peaks at 13.59°, 16.15°, and 17.75°, confirming the crystalline status of Dex whereas that of PLGA showed no peaks, indicating the amorphous state of the polymer. Comparably, the physical mixture showed peaks at the same 2θ values but with a lower intensity, revealing the presence of Dex crystals in the mixture diluted with the PLGA polymer. However, the Dex diffraction peaks were absent in the MP-Dex, which might be attributed to Dex’s molecular dispersion within the polymeric matrix. Broad halos, similar to the PLGA polymer’s diffractogram, were exhibited on examination of blank MP. Comparable patterns have been reported in the literature for Dex-loaded PLGA microparticles [45].

### 3.2. In Vitro Dexamethasone Release Study

**Figure 3:**
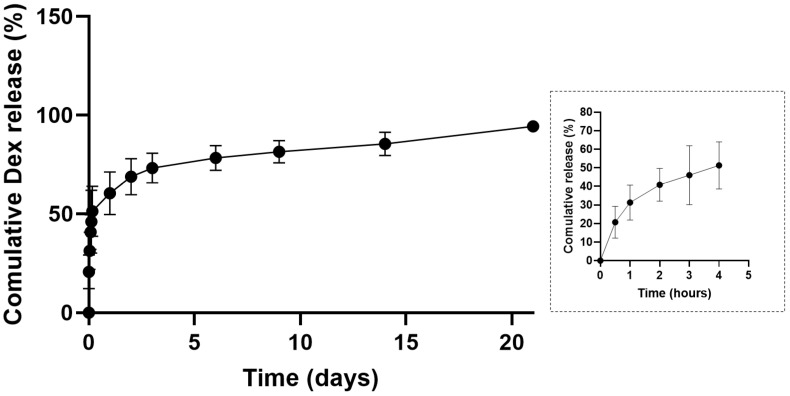
In vitro drug release profile of Dex from PLGA microparticles.

In vitro drug release was performed in PBS (pH 7.4) for MP-Dex for 21 days. As shown in Figure 3, the in vitro Dex release profiles displayed a burst release effect with 20.72% ± 8.57% released in 30 min, which indicates the drug was present at or near the surface of the microparticles. Generally, for PLGA microparticle-based systems, a triphasic drug release profile has often been observed [51]. From Figure 3, it was observed that the release was indeed following a three-phase pattern. After the initial fast release over the first 24 h, a controlled release pattern was observed over the next three days, which could be attributed to subsurface drug diffusion, followed by a gradual release of Dex from the slowly eroding PLGA matrix. At the end of the release study, on day 21, 95.30% ± 1.45% had been released. A similar pattern was previously reported for Dex release from PLGA microparticles prepared by the same method (the o/w emulsion solvent evaporation method) using a blend of PLGA grades [52]. In this study, therefore, loading the PLGA microparticles into a dissolvable MN system may be expected to initially offer a burst release followed by a controlled release of Dex over more than three weeks.

### 3.3. Microneedle Array Preparation and Characterisation

The MN array consisted of 3 × 3 conical needles, 900 µm in height and 450 µm in base diameter with a 1 mm tip-to-tip distance to allow enough space for the microparticles to diffuse into the surrounding tissue. The design dimensions are based on the previously reported finding that cone-shaped MNs show optimum geometry for tissue insertion and mechanical strength [53]. The array was fitted on a 4 × 4 mm square base fit onto the visible part of the sclera.

The MN array is largely composed of a dissolvable polymeric base; PVP and PVA are hydrophilic polymers that are commonly used in the preparation of ocular delivery dosage forms due to their favourable properties such as tear film stabilisation and lubrication. Blending PVP with PVA in the presence of water results in the formation of a chemical bond between the PVA hydroxyl group (–OH) and the proton acceptor carbonyl group on the pyrrolidone ring (–C=O) on PVP [54]. A PVP/PVA blend has been frequently used for the fabrication of dissolvable ocular MN arrays [8,11].

**Figure 4:**
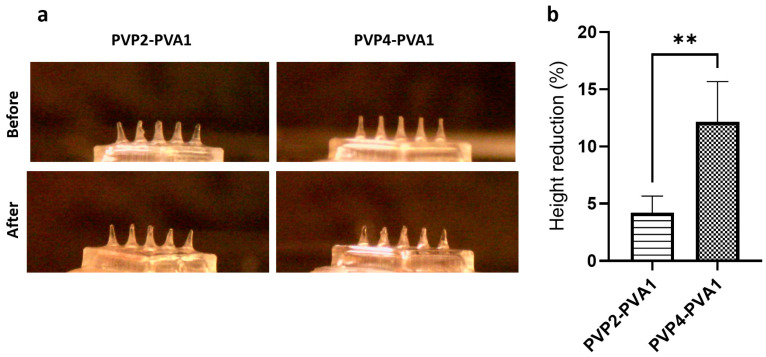
(**a**) Before and after images of MN array polymeric dissolution in gelatine prepared using different PVP/PVA blends (MNs are 900 µm in height before insertion). (**b**) Percentage of height reduction in MNs formulated using different PVP/PVA blends. Data are presented as the mean ± SD (*n* = 18) (** = *p* ˂ 0.001).

A blank MN array of 3 × 3 conical needles, 900 µm in height, and 450 µm in base diameter was used; the assembly is displayed in Figure 4a,b. Dissolution of the polymeric base was tested in gelatine to offer an environment that mimics ocular tissue water content [36]. Increasing the proportion of PVA from 25% (PVP4:PVA1) to 33.33% (PVP2:PVA1) resulted in a significant decrease in the height from 12.15% ± 2.52% to 4.20% ± 1.45% (Figure 4a,b); hence, increasing the PVA ratio prolonged the integrity of the needles, possibly via the greater preponderance of the interaction between the PVP and PVA molecules which in turn hindered the interaction with water [54]. A similar observation was reported for PVP/PVA gels, whereby a higher PVP concentration in the blend resulted in faster chain dissolution and lower matrix swelling [55].

**Figure 5:**
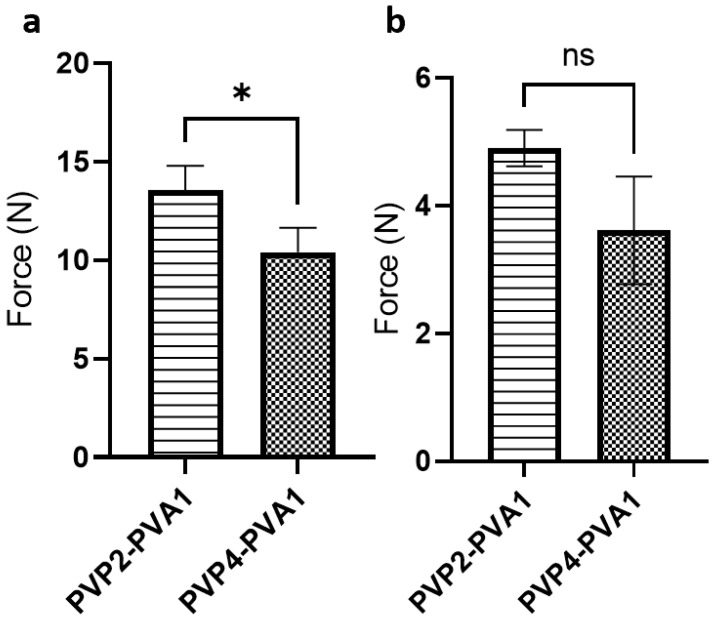
(**a**) Force exerted on MN arrays to reach 0.5 mm displacement. (**b**) Forces recorded to detach MN arrays prepared by various PVP/PVA blends from porcine scleral tissue. All values are presented as the mean ± SD (*n* = 3), (* = *p* < 0.05 and ns = not significant).

The mechanical strength of the two ratios of PVP/PVA blends was assessed by a compression test. A load was exerted perpendicular to the MN array and the force recorded at a displacement of 0.5 mm was measured (Figure 5a). Comparing the two formulations, PVP2-PVA1 showed a significantly higher compression force compared to PVP4-PVA1 (*p*-value ˂ 0.05); hence, the higher PVA ratio produced needles with greater mechanical robustness. Nevertheless, the mechanical strength of both MN array formulations indicated a structure sufficient to penetrate ocular tissue in accordance with the published literature [36].

The adhesion strength of the two PVP/PVA ratio systems on porcine scleral tissue was investigated using an Instron tensile tester. Generally, both PVA and PVP exhibit relatively weak mucoadhesive properties [56]. However, polymer properties such as molecular weight, moisture content, and hydrogen bonding capacity can affect the degree of adhesion to a biological tissue [57]. To assess the adhesion, a tensile force was exerted to withdraw the array after applying an initial compression force to attach the MN array to the scleral sample; the force required to detach the MN array from the tissue is considered to be the adhesion strength (Figure 5b). Comparing the two ratios, the higher PVA ratio system showed a numerically greater adhesion force, although this was not statistically significant (*p*-value > 0.05). Both PVP and PVA demonstrate a hydrogen-bonding ability that favours the interaction with the ocular mucin layer [58], as well as being of a molecular weight (greater than 100 kDa) commensurate with bioadhesive properties [59].

From the results of the dissolution, mechanical strength, and adhesion study, formula PVP2-PVA1 produced stronger needles with a slow dissolution and a relatively strong adhesion capability. Therefore, PVP2-PVA1 was selected to prepare the dissolving MN array for the rest of the study.

**Figure 6:**
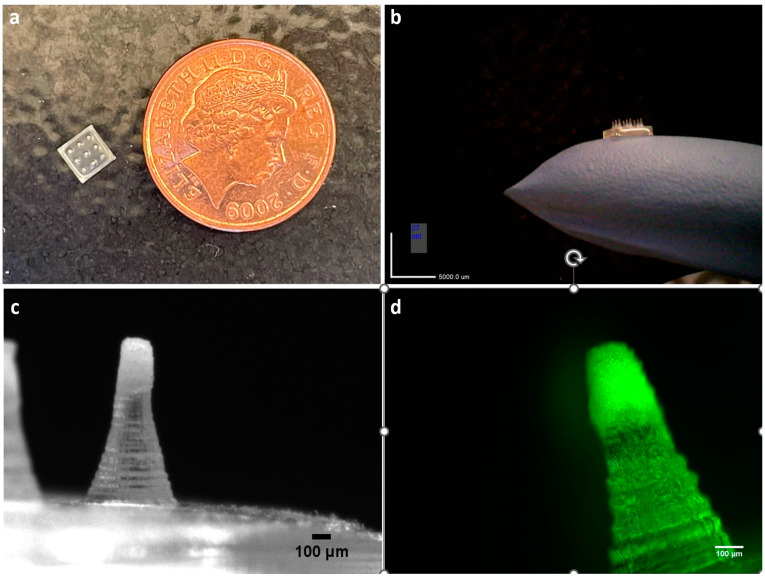
Digital images of MN array: (**a**) top view of unloaded MN array compared to a British penny (1p) coin (diameter = 20.3 mm), (**b**) side view, (**c**) MP-incorporated MN array concentrated at the tip, and (**d**) fluorescent microscope image of MP-Flu-incorporated MN array.

A range of microparticle-incorporated MN arrays were prepared. Blank microparticles (MN-MP), Dex-loaded microparticles (MN-Dex) and fluorescent labelled microparticles (MN-Flu) were mixed with 10% (*w*/*v*) P-K30 to cast the particles in the mould and left to dry under ambient conditions for 24 h. A PVP solution with a lower viscosity was used to cast the microparticles in the mould to attain a weak binding effect without hindering the release of the microparticles to the surrounding tissue upon insertion. The PVP/PVA blend (PVP2-PVA1) polymeric base solution was then added to the dried layer containing the microparticles and centrifuged. The fabrication process successfully concentrated the particles at the tip of the MNs, as shown in Figure 6c,d.

**Figure 7:**
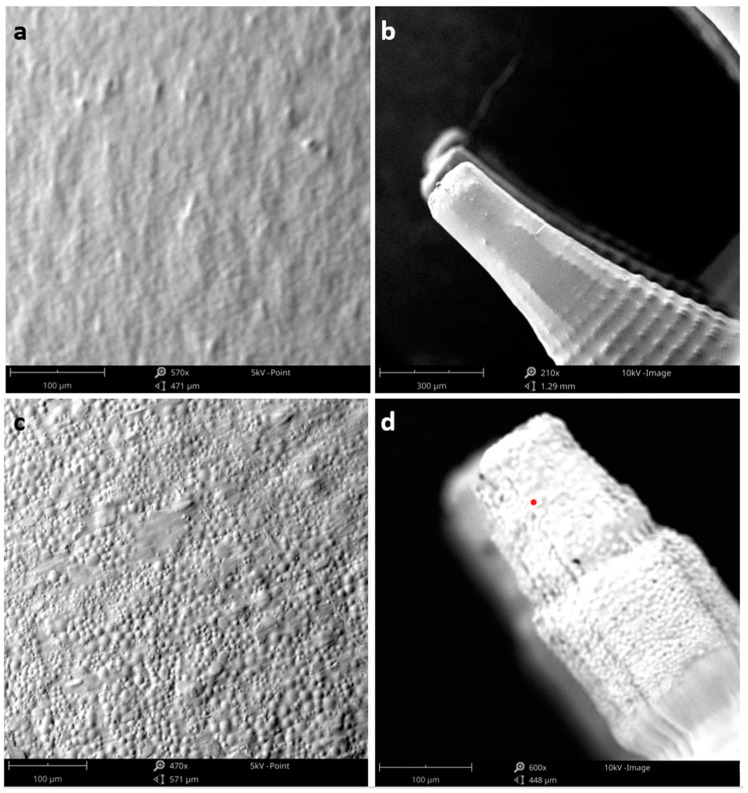
SEM images of MN array: (**a**) inter-needle space of unloaded MN array, (**b**) tip of the unloaded MN array, (**c**) inter-needle space of microparticle-incorporated MN array, and (**d**) MN tip of microparticle-incorporated MN array.

SEM images of the blank- and microparticle-incorporated MN arrays are presented in Figure 7. The inter-needle space and the MN tip are shown in Figure 7a,c and b,d, respectively. The blank MN tip and the inter-needle space displayed a smooth surface, while images of microparticles incorporated in MN arrays confirmed the concentration of the particles at the tip of the needles. Examination of the inter-needle space revealed that particles were found on or near the surface of the MN base, indicating that a proportion of the microparticles did not travel to the tip of the MN during centrifugation. However, the microparticles in the inter-needle space may nevertheless offer episcleral delivery at the insertion site.

**Figure 8:**
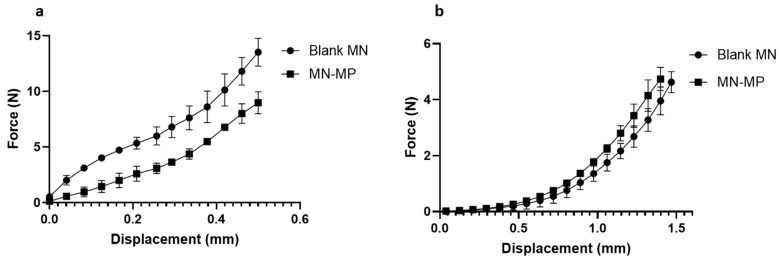
MN array (3 × 3) (**a**) mechanical strength test of blank MN array and MN-MP, and (**b**) ex vivo scleral insertion test of blank MN array and MN-MP. The data are presented as the mean ± SD (*n* = 3).

The microparticle-incorporated MN array (MN-MP) was tested for mechanical strength to evaluate the effect of the microparticle addition. Figure 8a depicts the mechanical strength of a blank MN array and the MN-MP. The addition of particles significantly (*p*-value ˂ 0.05) reduced the compression resistance from 13.53 N ± 1.25 per array (1.5 N/needle) to 8.99 N ± 0.98 per array (0.99 N/needle), which is consistent with the data reported in the literature [36,60]. This could be attributed to the low packing density of the microparticles at the MN tip and the lower viscosity grade of PVP that was used to suspend the particles before centrifugation.

The insertion capability of a polymeric and microparticle-loaded MN array into the ocular tissue was estimated from the compression distance vs. force curve [36]. The MN array insertion force–distance curve into scleral tissue is shown in Figure 8b. The force required to penetrate the sclera was 0.774 ± 0.241 N/array (0.086 N/needle) for the blank MN array and 0.782 ± 0.246 N/array (0.087 N/needle) for the MN-MP. In both cases, blank MN and MN-MP arrays showed no significant difference (*p*-value ˂ 0.05) in the forces required to penetrate the scleral surface, reflecting the consistent mechanical resistance of the sclera itself. More importantly, the penetration forces recorded were small enough to insert the MN array into the sclera by gentle thumb pressure (~4 N) [36]. Incorporating MPs into the MN array slightly increased the force required for insertion; this could be due to the lower needle strength of MN-MP recorded in the mechanical strength test. A limited number of previous studies have investigated the penetration force of dissolvable MNs into scleral tissue [7,9,10], while other studies have reported corneal insertion forces [7,8,11,13,36,61]. The penetration force is highly influenced by several factors including the material used, needle geometry, and tip size [62]. More specifically, one study has reported an 11 × 11 MN array prepared by PVP K30 whereby a 0.08 N/needle force was recorded for scleral insertion [9], while another tested the force required to fully insert a 3 × 3 MN array of 800 µm in height and fabricated using a PVP K29-32 polymeric base [7]. Insertion forces of 0.148 N/ needle on porcine scleral tissue and a three times higher force of 0.413 N/needle for corneal tissue for full MN penetration were recorded.

### 3.4. Ex Vivo Transscleral Permeability

**Figure 9:**
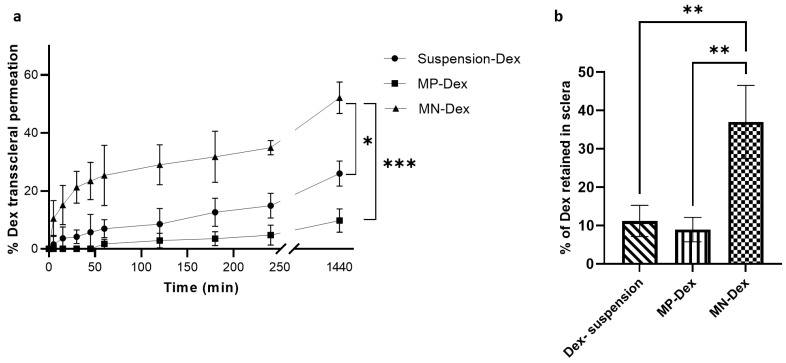
(**a**) In vitro transscleral permeation profile of Dex from Dex suspension, Dex loaded microparticles (MP-Dex) and MP-Dex incorporated MN array (MN-Dex), and (**b**) percentage of Dex retained in sclera after the permeation study. Values are presented as the mean ± SD, (* = *p* < 0.01, ** = *p* < 0.001, and *** = *p* < 0.0001).

The transscleral Dex permeation profile is presented in Figure 9a. Dex permeation was significantly higher in the MN-Dex compared to free (i.e., not incorporated into MNs) MP-Dex and Dex suspension. At 4 h after application, 34.93% ± 2.44% permeated from MN-Dex, 14.89% ± 4.26% from Dex suspension, and 4.75% ± 3.45% from MP-Dex. At 24 h, Dex permeation was 45.12% ± 5.39% from MN-Dex, 25.96% ± 4.35% from Dex suspension, and 9.75% ± 4.06% from free MP-Dex. This significant difference between MN-Dex and the other dosage forms is attributed to the fact that MNs pierce the tissue surface, partially overcoming the scleral barrier, while Dex suspension permeation was greater than the free MP-Dex, as expected. Dex is able to demonstrate some scleral permeation in all delivery forms, as previous studies have indicated that the relatively porous scleral membrane allows passage of molecules up to 150 kDa [3]. Nevertheless, the well-recognized anatomical and physiological barriers to drug molecules reaching the vitreoretinal region [63] means that conventional topical drug administration achieves a low level of delivery to the inner tissues [64]. The current and previous studies have demonstrated that MN systems may represent a means of significantly increasing the delivered concentration of a drug compared to free drug application approaches [32].

The amount of Dex retained in the sclera was significantly higher (*p*-value < 0.001) in the case of MN-Dex as compared to the free MP-Dex and Dex suspension (Figure 9b). This is potentially due to MN-Dex tissue penetration and microparticle deposition within the scleral tissue. A similar finding was reported with the corneal application of a besifloxacin-loaded PVP/PVA dissolvable MN array, whereby the amount of besifloxacin detected in the cornea post MN application was almost four times higher than the amount detected after topical solution instillation [11].

### 3.5. Ex Vivo Microparticle Diffusion

**Figure 10:**
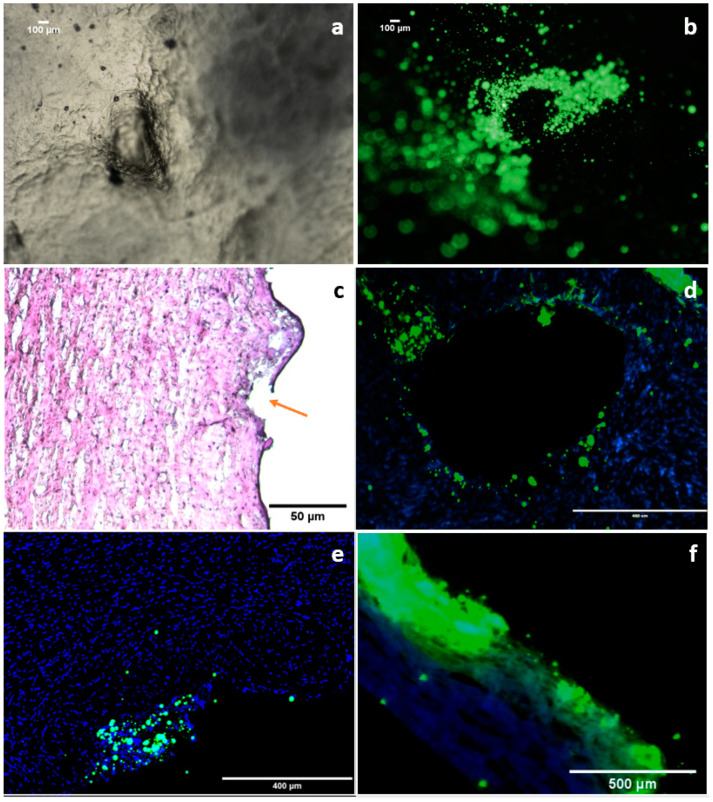
Images of ex vivo particle diffusion: (**a**) light microscopy image of MN around the insertion point of the scleral surface, (**b**) fluorescence microscopy image of MP-Flu release on the scleral surface, (**c**) H&E stain of cryosection sclera obtained after insertion of MN (the orange arrow indicates the crack wound), (**d**) fluorescence microscopy images of middle-region transverse sections of ex vivo MN-Flu diffusion study 1 h after insertion, (**e**) confocal microscopy image of cross-sectioned sclera 1 h after MN array is loaded with MP-Flu insertion, and (**f**) fluorescence microscopy images’ cross-sectioned sclera 2 h after insertion.

Fluorescent-labelled microparticles (Flu-MP) were used to track the diffusion of the microparticles in porcine scleral tissue. Porcine eyes represent a suitable model to study ocular diffusion, as porcine sclera has a high degree of homology with human sclera compared to other animal models and also a resemblance to the human sclera in terms of water content, histological structure, and collagen bundle arrangement [65]. Figure 10a–c shows that the MN has created holes on the surface of the scleral tissue and released the MP-Flu around the insertion crack, indicating the dissolution of the MN tip. In Figure 10d, the transverse section of scleral tissue 1 h after insertion reveals the diffusion of Flu-MP into the surrounding tissue. Images of cross-sectioned scleral tissue after insertion with MN-Flu for 1 and 2 h have further confirmed microparticle diffusion, showing that MP-Flu has transversely diffused within the collagen and elastin fibres within the scleral matrix.

The sclera is a porous membrane with water-filled channels between collagen bundles. Thus, passive diffusion may be the main method of transport into the sclera, with active transport being considered improbable [66]. The data indicate that smaller particles (˂500 nm) can diffuse through scleral channels and suprachoroidal space due to the small spacing between the collagen fibre in the sclera, whereas larger-particle (˃500 nm) flow between the collagen fibre is more constrained due to the scleral sieving effect [67]. A previous study has compared the delivery of microparticles and nanoparticles to the suprachoroidal space using a solid hollow microneedle; the nanoparticles showed a significant transscleral diffusion whereas the microparticles remained trapped in the suprachoroidal space [67]. A further study has similarly reported the delivery of PLGA nanoparticles to the scleral tissue using dissolving MNs. The nanoparticles displayed a considerable increase in scleral penetration using dissolvable MNs compared to topical administration [14]. There is not enough evidence in the literature to support the hypothesis that microparticles travel across the sclera and reach the retinal layer. However, with restricted particle diffusion into the deeper scleral layer, particle retention and localisation in the scleral matrix can offer a drug delivery depot where a drug can be released from the particles and permeate to the inner tissue.

It is worth noting that the scleral matrix is primarily composed of negatively charged collagen bundles, making it more permeable to anionic molecules [68]. In this study, the fabricated MP-Flu have a negative surface charge which implies that the particles tend to be less aggregated, and thus more flexible to spread between the collagen bundles. Moreover, the spherical shape of the prepared microparticles could be advantageous in improving particle diffusion, with shape also having been reported to influence particle permeability [69]. Furthermore, globular-shaped and negatively charged macromolecules have been demonstrated to exhibit high permeability across the sclera compared to positively charged linear or branched macromolecules [65]. It was also reported that the ocular epithelial layer is a major barrier to particle penetration; hence, methods to disrupt the integrity of this layer have been employed to improve particles’ ocular permeation [38]. The physical disruption of the ocular surface using MNs is a possible route to overcome this, although this was not explored in the present study.

### 3.6. Dexamethasone Ocular Biodistribution

**Figure 11:**
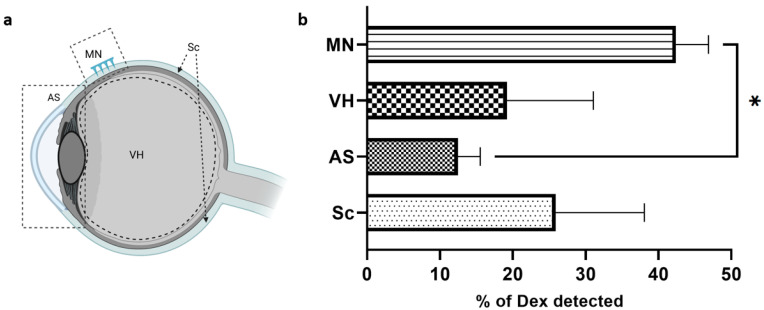
Dex ocular diffusion: (**a**) the regions of Dex detection in the ocular globe, and (**b**) percentage of Dex detected in each region of the eye (Sc = sclera, AS = anterior segment, VH = vitreous humour, MN = MN insertion site). Data are presented as the mean ± SD, (* = *p* < 0.01).

This study was designed to investigate Dex diffusion within the ocular globe. A MN array loaded with MP-Dex was inserted on an intact porcine eye. The eye globe in the experiment was subsequently segmented into the anterior segment (AS), vitreous humour (VH), MN array insertion region (MN), and the rest of the posterior segment (Sc) (Figure 11a). The total amount of Dex found was used as the comparative reference to calculate the percentage detected in each part of the eye. The total percentage of Dex detected in the eyes based on the drug loading estimation in each MN array was 79.6% ± 14.3% for the MN-Dex array. This percentage may potentially be raised by increasing the application time or adding an insoluble backing layer to help maintain the array at the required site.

The highest percentage of Dex detected was at the site of MN insertion (42.4% ± 4.5%), which was expected due to direct particle deposition into the scleral matrix. However, this figure is a net amount of Dex released and remaining encapsulated in the deposited particles. However, the major challenge in treating posterior ocular diseases is delivering an effective dose that overcomes the ocular barrier and reaches the target tissue [3]. The data, in this respect, are encouraging, with 19.2% ± 11.9% with MP-Dex found in the VH after 4 h. Moreover, the drug content in the rest of the sclera (excluding the insertion site), the choroid, and the retina was 25.9% ± 12.2%. This may be attributed to drug diffusion, and possibly particulate transverse diffusion; as mentioned above, the scleral collagen fibres are known to have a lateral orientation that facilitates such mobility [65]. The lowest amount of Dex was detected in the AS with 12.48% ± 3.05%, which could reflect diffusion through the episcleral surface and aqueous fluid surrounding the eye globe.

## 4. Conclusions

In this investigation, a dissolvable polymeric MN array incorporating biodegradable PLGA microparticles for transscleral delivery was developed and the ex vivo behaviour was studied. Various PVP/PVA blends were tested as MN fabrication materials to optimise the dissolution, strength, and adhesion of the polymeric base of the MNs. The dissolvable MN array displayed sufficient strength to penetrate the sclera; the insertion force was determined and found to be small enough to pierce the sclera by gentle pressure. Finally, to determine transscleral particle distribution, fluorescently labelled microparticles incorporated into the MN array were inserted into the sclera. The dissolvable MN system successfully deposited the microparticles, with transactional images confirming that the microparticles diffused through the scleral matrix. Additionally, Dex was detected in the vitreous humour after the application of MNs. The findings indicate the potential of the platform to deliver Dex to the posterior segment of the eye. Further assessment of the novel delivery approach, including in vivo assessment, may expand the understanding of the potential capabilities of the system.

## Figures and Tables

**Table 1:** Composition of 30% *w/v* base solutions used to prepare MN-array polymers expressed as mass of PVP and PVA in 100 mL of solution.

Formulation Name	PVP	PVA
PVP4-PVA1	24 mg	6 mg
PVP2-PVA1	20 mg	10 mg

**Table 2:** Characterisation of PLGA microparticles (blank, Dex-loaded, and fluorescent-labelled).

Formulation Name	Size (d.nm)	ζ Potential (mV)	%*DL*	%*EE*
Blank MP	2690 ± 596	−13.8 ± 4.7	-	-
MP-Dex	1860 ± 215	−11.3 ± 2.4	14.3 ± 1.9	49.4 ± 7.3
Flu-MP	2647 ± 169	−21.5 ± 0.4		

## Data Availability

The data presented in this study are available on request from the corresponding author.
